# Examining disparities in cardiovascular disease prevention strategies and incidence rates between urban and rural populations: insights from Kazakhstan

**DOI:** 10.1038/s41598-023-47899-8

**Published:** 2023-11-28

**Authors:** Natalya Glushkova, Botagoz Turdaliyeva, Maksut Kulzhanov, Indira K. Karibayeva, Maksut Kamaliev, Dariga Smailova, Ayaulym Zhamakurova, Zhanar Namazbayeva, Gulmira Mukasheva, Asylzhan Kuanyshkalieva, Nurzhamal Otyzbayeva, Yuliya Semenova, Bagym Jobalayeva

**Affiliations:** 1 Department of Epidemiology, Evidence-Based Medicine and Biostatistics, Kazakhstan’s Medical University “Kazakhstan School of Public Health”, Almaty, 050060 Kazakhstan; 2 Kazakh Scientific Center of Dermatology and Infectious Diseases, Almaty, 050002 Kazakhstan; 3 Department of Management in Healthcare and Pharmacy, Kazakhstan’s Medical University “Kazakhstan School of Public Health”, Almaty, 050060 Kazakhstan; 4 Department of Science and Consulting, Kazakhstan’s Medical University “Kazakhstan School of Public Health”, Almaty, 050060 Kazakhstan; 5 Department of Health Management, Kazakhstan’s Medical University “Kazakhstan School of Public Health”, Almaty, 050060 Kazakhstan; 6 Research Department, Asfendiyarov Kazakh National Medical University, Almaty, 050040 Kazakhstan; 7 Department of Postgraduate Education, Kazakhstan’s Medical University “Kazakhstan School of Public Health”, Almaty, 050060 Kazakhstan; 8 School of Medicine, Nazarbayev University, Astana, 010000 Kazakhstan; 9 Department of Public Health, JSC “Semey Medical University”, Semey, 071400 Kazakhstan

**Keywords:** Cardiology, Diseases, Health care

## Abstract

Kazakhstan is experiencing a high burden of cardiovascular disease (CVD), and the country has implemented a range of strategies aimed at controlling CVD. The study aims to conduct a content analysis of the policies implemented in the country and augment it with an analysis of official statistics over a 15-year period, from 2006 to 2020. The study also includes comparisons of incidence rates between urban and rural areas. A comprehensive search was conducted to identify policy documents that regulate the provision of primary, secondary, and tertiary prevention of cardiovascular diseases. Additionally, official data on the incidence of arterial hypertension, ischemic heart disease, acute myocardial infarction, and cerebrovascular disease were extracted from official statistics, disaggregated by urban and rural areas. Forecast modeling was utilized to project disease incidences up to 2030. The study reveals that Kazakhstan primarily focuses on tertiary prevention of cardiovascular diseases, with less attention given to secondary prevention, and primary prevention is virtually non-existent. In general, screening for arterial hypertension appears to be more successful than for ischemic heart disease. The incidence of arterial hypertension has increased threefold for urban residents and 1.7-fold for rural residents. In urban areas, residents saw a twofold increase in ischemic heart disease incidence, while it remained the same in rural areas. The findings of this study have practical implications for decision-makers, who can use the results to enhance the effectiveness of existing CVD prevention strategies.

## Introduction

Cardiovascular disease (CVD) is a significant global health challenge that affects individuals, families, communities, and societies as a whole. The World Health Organization (WHO) reports that CVD is the leading cause of death worldwide, with a staggering 17.9 million deaths recorded in 2019^1^. Despite advancements in medical research and technology, CVD continues to be the leading cause of mortality in many low- and middle-income countries. This is often attributed to insufficient implementation of existing preventive and treatment strategies^2^. Notably, the countries of the former Union of the Soviet Socialist Republic (USSR) have been severely affected by the burden of CVD, with ischemic heart disease (IHD) ranking as the leading cause of death from non-communicable diseases (NCD), while stroke ranks second^3^. This highlights the urgent need for effective and sustainable measures to prevent and control CVD.

Furthermore, it is crucial to acknowledge that the impact of CVD extends beyond mortality and significantly affects the quality of life of individuals with the disease. Thus, prioritizing the prevention and control of CVD is imperative to mitigate its adverse effects on individuals, families, communities, and society as a whole. This necessitates comprehensive strategies that encompass prevention, early detection, diagnosis, treatment, and management of CVD. Achieving this goal requires collaboration among various stakeholders, including governments, healthcare providers, researchers, and individuals themselves, to reduce the burden of CVD and promote better health outcomes for all^1^.

The prevention of CVD can be categorized into three levels: primary, secondary, and tertiary prevention. Primary prevention involves lifestyle modifications such as adopting a healthy diet, engaging in physical activity, and quitting smoking. Secondary prevention is crucial in reducing the burden of CVD, as it involves identifying individuals who already have the disease and providing them with appropriate medical care to prevent disease progression. Tertiary prevention entails providing individuals with CVD complications timely and appropriate medical and rehabilitation services to improve their physical and mental well-being and prevent premature death. It is imperative for each country to have all preventive strategies in place^4^.

The Republic of Kazakhstan (hereafter—Kazakhstan) is a Central Asian state that gained independence in 1991 after the breakup of the USSR. Like other countries in the region, Kazakhstan faces a high burden of CVD. To address this issue, the country has implemented a range of public health strategies aimed at reducing CVD-associated mortality. While existing scientific literature has documented the effectiveness of these strategies^5–7^, there is currently no research that considers the continuum of preventive measures as a whole. To bridge this gap, the present study intends to perform a content analysis of the policies implemented by Kazakhstan, and augment it with an analysis of official statistics on CVD. Moreover, the research will investigate the variations in CVD prevention approaches and occurrence rates among urban and rural communities, taking into account the probable disparities in healthcare delivery. The findings of this study have practical implications for decision-makers, who can use the results to enhance the effectiveness of existing CVD prevention strategies.

## Materials and methods

**Figure 1:**
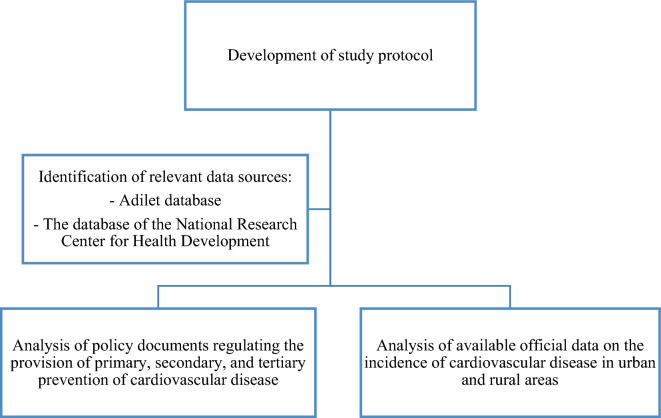
The study flowchart.

This study consists of two distinct yet coherent stages. In the first stage, a comprehensive search was conducted for policy documents that regulate the provision of primary, secondary, and tertiary prevention of CVD to the population of Kazakhstan. These findings were then supplemented by an analysis of available official data on CVD incidence to evaluate the effectiveness of existing prevention strategies. Figure 1 presents the study flowchart.

### Analysis of policy documents regulating the provision of preventive strategies for CVD

A series of searches were conducted to locate policy documents related to the provision of preventive strategies for CVD. Given that official documents in Kazakhstan are typically published on the Adilet database, which is maintained by the Ministry of Justice, this was the initial source consulted in all searches. Additionally, the National Research Center for Health Development, named after Salidat Kairbekova, was also consulted as this database contains healthcare policy documents. Finally, a Google search was conducted to identify any remaining evidence pertaining to national health policies on CVD prevention.

Since the population of Kazakhstan is bilingual, searches were conducted in both Kazakh and Russian languages. For searches in Russian, the following search queries were used: (i) 'пpoфилaктикa' (meaning 'prevention'), (ii) 'пepвичнaя' (meaning 'primary'), (iii) 'втopичнaя' (meaning 'secondary'), (iv) 'тpeтичнaя' (meaning 'tertiary'), (v) 'cepдeчнo-cocyдиcтыe зaбoлeвaния' (meaning 'cardiovascular disease'), and (vi) 'Кaзaxcтaн' (meaning 'Kazakhstan'). For searches in Kazakh, the following keywords were considered: (i) 'aлдын aлy' (meaning 'prevention'), (ii) 'бacтaпқы' (meaning 'primary'), (iii) 'қocaлқы' (meaning 'secondary'), (iv) 'үшiншi' (meaning 'tertiary'), (v) 'жүpeк-қaн тaмыpлapы aypyлapы' (meaning 'cardiovascular disease'), and (vi) 'Қaзaқcтaн' (meaning 'Kazakhstan'). The titles of all identified documents were reviewed, and relevant documents in both languages were listed. Duplicates were removed after comparing the two lists. Finally, the full-text versions of the remaining documents were obtained, and content analysis was conducted by extracting data into specially designed tables.

### Analysis of cardiovascular disease incidence in urban and rural areas at the national and regional levels

For this analysis, we retrieved official data collected by the Ministry of Health (MoH). The MoH releases national healthcare statistics annually in the form of a statistical yearbook titled “Public Health in the Republic of Kazakhstan and Activities of Healthcare Facilities”. This compilation is based on reports submitted by Kazakhstan’s regional health authorities. During the study period (2006–2020), 14 administrative regions, locally known as “oblasts” (Aktobe, Mangystau, Atyrau, South Kazakhstan, Zhambyl, Kyzylorda, Almaty, West-Kazakhstan, Akmola, Karaganda, East Kazakhstan, Kostanay, North Kazakhstan, and Pavlodar), were included. Electronic versions of the statistical compilation for the period 1999–2020 are freely accessible on the website of the National Research Center for Health Development^8^. However, information on urban–rural disaggregation in the incidence of CVD is only available beginning in 2006. Details of research carried out with the help of this statistical compilation are presented elsewhere^9,10^.

For subsequent analysis, we extracted information from the "Health Indicators" subsection of this year's statistical report. All indicators are already presented as incidence rates per 100,000 of the respective population. The incidence of (CVD is reported as a whole and disaggregated by different diagnostic units: arterial hypertension (AH), ischemic heart disease (IHD), acute myocardial infarction (AMI), and cerebrovascular disease. In Kazakhstan, cerebrovascular disease mainly comprises stroke and transient ischemic attacks and was included in the analysis as a CVD complication. Only incidence rates for the adult population were extracted, defined as individuals aged 18 years and above, for the purposes of this study. The dataset used for the analysis is presented in Appendix A.

### Statistical analysis

In this study, we utilized Statistical Package for Social Sciences (IBM SPSS Statistics) version 24.0 to conduct forecast modeling. Specifically, we used the Expert Modeler function of this statistical software to auto-detect the best-fit epidemiological model for predictive analysis. To project the incidences of the studied diseases (AMI and cerebrovascular disease) for the period up to 2030, we utilized the observed incidences from 2006 to 2020, and reported the projections with 95% confidence intervals (CI). We extracted the parameters of the best-fit model and set the significance of all tests to p = 0.05. To visualize the regional variations in the incidence of AH and IHD, we created maps of Kazakhstan using QGIS 3.26 “Buenos Aires”. Algorithms for the statistical analysis are presented in Appendix B.

### Ethics statement

This study received a permission of the Ethics Committee of Semey Medical University (Protocol #2 of 28.10.2020).

## Results

Table 1 provides an overview of the activities related to primary, secondary, and tertiary prevention of CVD in Kazakhstan, along with the regulatory standards in place. While clinical practice guidelines are widely recognized as the best policy documents for regulating prevention measures, we were unable to find any specific to Kazakhstan despite conducting extensive searches. Furthermore, we were unable to locate any policies targeting primary prevention of CVD. The only mention of primary prevention was in the MoH order that approved regulations for hospitals and clinics offering cardiac, interventional cardiac, and cardiac surgical treatments^11^. The order stated that "coordination, organization, and implementation of primary and secondary prevention activities for CVD should be provided by cardiology centers." However, the order did not provide details on the scope of these activities. We did find a clinical protocol for the diagnosis and treatment of patients with acute coronary syndrome, which outlined preventive measures such as smoking cessation, diet and exercise, and blood pressure (BP) control (with a recommended systolic BP not exceeding 140 mmHg and diastolic BP not exceeding 90 mmHg). Although these measures are typically associated with primary prevention, the protocol only recommends them for patients who have already been diagnosed with the disease^12^.

**Table 1:** Provision of activities related to primary, secondary, and tertiary prevention of cardiovascular disease.

Type of prevention	Aim	Internationally recognized regulatory standard	Regulatory standard available in Kazakhstan, reference
Primary	Prevent CVD from occurrence in populations at risk	Clinical practice guidelines	No standard has been established for this type of prevention
Secondary	Early identification and appropriate treatment of CVD	Clinical practice guidelines	1. The MoH order approving the regulations for conducting preventive medical examinations among specific target population groups^13^ 2. The MoH order approving the standard of cardiac, interventional cardiac, and cardiac surgical care^14^ 3. Clinical protocols for diagnosis and treatment of patients with established CVD^15^
Tertiary	Treatment of complications associated with CVD	Clinical practice guidelines	1. The MoH order approving the standard of cardiac, interventional cardiac, and cardiac surgical care^14^ 2. The MoH order approving the regulations for hospitals and clinics that provide cardiac, interventional cardiac, and cardiac surgical treatment^11^ 3. Clinical protocols for diagnosis and treatment of patients with acute myocardial infarction, acute coronary syndrome, and stroke^15^

*CVD* cardiovascular disease, *MoH* Minister of Health.

Currently, in Kazakhstan, two types of screening programs are available for CVD: one targeting AH and the other focusing on IHD. The MoH has established regulations for conducting preventive medical examinations among specific target population groups, which determine the scope of procedures related to the provision of these screening programs^13^. The screening is intended for individuals aged 40 years and older and is conducted biennially. Both screening programs occur simultaneously and are divided into two stages. In the first stage, a nurse examines the patient, conducts interviews, and makes all specified measurements. In the second stage, a doctor interprets the test results and evaluates the patient's CVD risks, providing recommendations accordingly. Further details regarding the provision of CVD screening in Kazakhstan are available in Table 2.

**Table 2:** Provision of screening programs for cardiovascular disease in Kazakhstan^13^.

Screening stage, medical personnel involved	Venue	Target population	Related activities
First stage, nurse	Primary healthcare facility	Individuals between the ages of 40 and 70, regardless of gender, are advised to undergo biennial screenings	1. Measurements of weight, height, and waist circumference, calculation of body mass index
			2. Conducting a questionnaire survey^a^
			3. Measurements of the blood pressure, with a 1–2 min interval, while seated
			4. Performance of total cholesterol test
Second stage, primary practitioner	Primary healthcare facility		1. Utilization of the SCORE^b^ chart to assess a patient's cardiovascular risk and determine their further management:
			1A. A follow-up examination after two years for individuals with low cardiovascular risk (up to 1% on the SCORE chart) and the importance of maintaining a healthy lifestyle to sustain a low cardiovascular risk is emphasized
			1B. Individuals with a moderate risk of cardiovascular disease (defined as having a SCORE chart score of greater than 1% but less than 5%) are recommended to attend a health program, with the aim of mitigating or stabilizing their cardiovascular risk
			1C. Individuals with high and very high cardiovascular risk (defined as having a SCORE chart score of greater than 5%) are recommended to undergo electrocardiography (ECG). If any abnormalities are detected on the ECG, or if the individual has a cholesterol level of over 5.0 mmol/L, blood pressure above 140/90 mmHg, or a heart rate outside of the normal range, they are referred to an internist for further evaluation
			1D. If a patient is diagnosed with cardiovascular disease (CVD), the condition is managed appropriately. For individuals without CVD, they are directed to a health program aimed at reducing their risk of developing cardiovascular disease. If deemed necessary, a patient may be referred to a cardiologist for further consultation

^a^The type of questionnaire to be used has not been specified.

^b^SCORE—Systematic COronary Risk Evaluation Chart.

**Figure 2:**
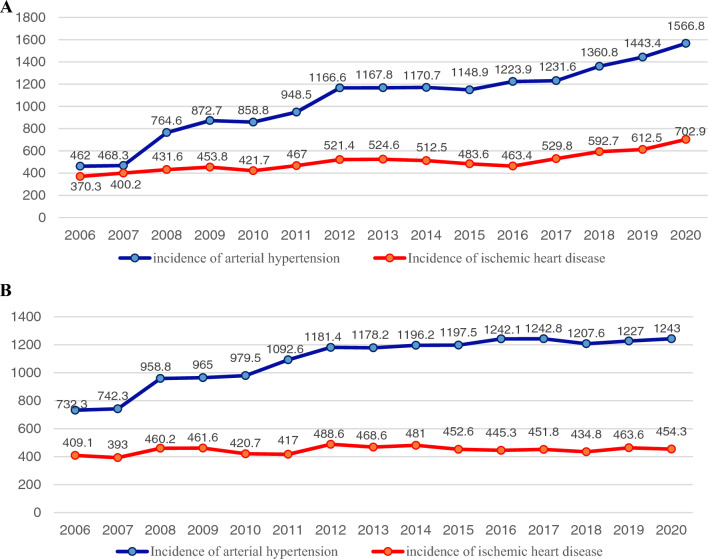
Incidence rates of arterial hypertension and ischemic heart disease in urban (**A**) and rural (**B**) areas of Kazakhstan, 2006–2020.

Figure 2A and B provide evidence for the effectiveness of screening programs targeting AH and IHD in Kazakhstan, as they illustrate the 15-year incidence trends for urban and rural areas, respectively. It is commonly assumed that increasing the screening effectiveness leads to higher incidence rates^16^. According to Fig. 1, the detection rates for AH and IHD are higher in urban areas. Specifically, the incidence of AH in urban areas tripled from 462 per 100,000 population in 2006 to 1566.8 in 2020, while the incidence of IHD increased more modestly from 370.3 per 100,000 in 2006 to 702.9 in 2020. In contrast, in rural areas, the increase in the incidence of AH was less pronounced, at 1.7-fold (from 732.3 per 100,000 population in 2006 to 1243 in 2020), and there was no change in the incidence of IHD, as the respective time series appears to be stationary throughout the entire observation period. These findings suggest that screening for IHD is less effective than for AH in Kazakhstan, particularly in rural areas.

**Figure 3:**
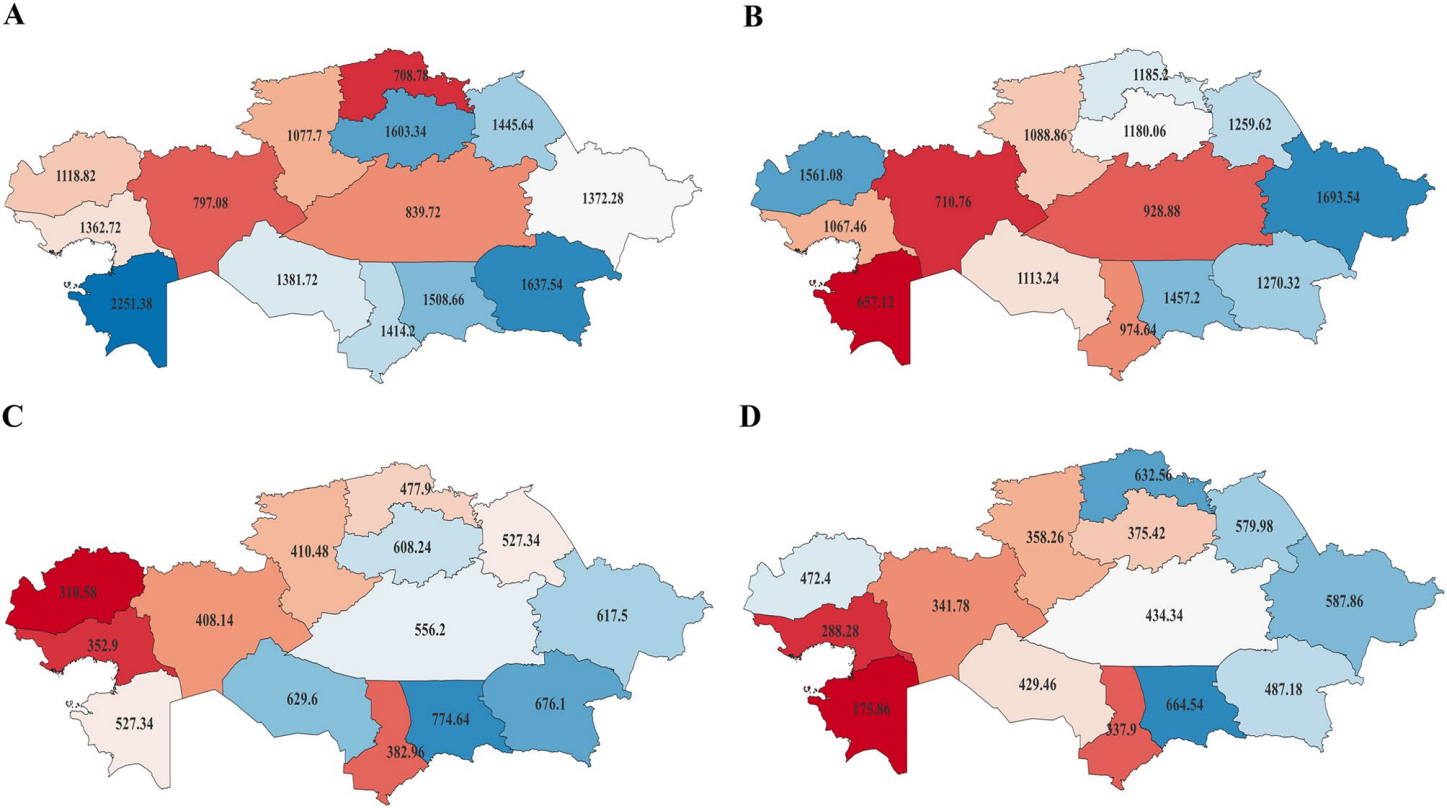
A map of Kazakhstan's regions, illustrating the distribution of mean annual incidence rates of arterial hypertension in urban (**A**) and rural (**B**) areas, as well as ischemic heart disease in urban (**C**) and rural (**D**) areas, during the period of 2016–2020, with rates expressed per 100,000 population.

Figure 3 provides insights into regional variations in the incidence of AH and IHD over the past five years (2016–2020). The color code used in the figure indicates that dark red shades correspond to the lowest incidence rates, while dark blue shades represent the highest. This figure can be used to illustrate the variations between urban and rural regions in the effectiveness of screening programs. In particular, the urban areas of North Kazakhstan region had the lowest incidence rates of AH in the country (708.78 per 100,000 population), while those of Mangystau region had the highest (2251.38 per 100,000 population). However, the patterns are different when considering rural regions. Mangystau region had the lowest incidence rates of AH in the country (657.12 per 100,000 population), while the incidence of AH in rural North Kazakhstan region was above the country's average. These disparities in urban–rural AH incidence may be attributed to different levels of screening effectiveness.

Regarding the incidence of IHD, there was more consistency in the urban–rural variations, as shown in Fig. 3C and D. For example, the population of Zhambyl region had the highest IHD rates in the country in both urban (774.64) and rural areas (664.54). Additionally, the urban population of Atyrau region had the second-lowest IHD incidence in the country (352.9), while the rural population had the lowest incidence (288.28).

Table 3 presents a summary of policy documents that require managing patients with CVD complications, which are considered measures of tertiary prevention. As the table illustrates, Kazakhstan has made significant strides in improving medical care for this patient population, implementing regulations aimed at enhancing the availability and accessibility of care and providing substantial funding. Moreover, it is worth noting that all interventional cardiac and cardiac surgical treatments are fully covered by the government, including patient transportation to specialized healthcare facilities^17^. Overall, these healthcare facilities are equipped according to international standards, and healthcare personnel receive ongoing qualification upgrades, including attachments to leading surgical centers abroad. Furthermore, since 2020, interventional cardiac departments have been established in all multidisciplinary central district hospitals^14^, making percutaneous coronary interventions more accessible to rural residents.

**Table 3:** Legislative acts mandating the provision of tertiary prevention to patients with cardiovascular disease.

Type of legislative act, reference	The initial year of implementation and any subsequent updates	Key strategies for tertiary prevention of CVD
The MoH order approving the standard of cardiac, interventional cardiac, and cardiac surgical care^14^	2016 2017 2021	The order describes the procedures for providing planned and emergency medical aid to patients with CVD, outlining the steps for effectively managing acute and chronic CVD-related conditions The order outlines the regionalization of healthcare facilities that provide care for CVD, as well as the appropriate level of surgical intervention to be offered at each type of facility
The MoH order approving the regulations for hospitals and clinics that provide cardiac, interventional cardiac, and cardiac surgical treatment^11^	2011	The order prescribes the structural and operational requirements for healthcare organizations providing cardiac, interventional cardiac, and cardiac surgical care to the population of Kazakhstan, defining the scope of activities for each type of facility
The MoH order approving the minimum standards for medical device equipment in healthcare organizations^18^	2020 2022	The order outlines the specific medical equipment to be provided to various types of healthcare facilities that offer cardiac, interventional cardiac, and cardiac surgical services. The list of diagnostic and therapeutic equipment is provided separately, with particular emphasis placed on equipment for surgical and intensive care units
The MoH order approving the minimum standards for healthcare personnel working in healthcare organizations^19^	2010–2013 2017–2020 2023	The order outlines the distribution of healthcare personnel specializing in various cardiology subspecialties across healthcare facilities located in rural, urban, and national settings, based on the number of population
Clinical protocols for diagnosis and treatment of patients with acute myocardial infarction, acute coronary syndrome, stroke^15^	2016 2017	The protocols delineate the diagnostic and treatment modalities to be administered to a patient with acute myocardial infarction, acute coronary syndrome, or stroke, including the pharmaceuticals, interventions, and surgeries to be employed, as well as their corresponding indications and contraindications

*CVD* cardiovascular disease.

Kazakhstan has made significant efforts to improve the tertiary prevention of cardiovascular disease (CVD) by mobilizing substantial financial and human resources. It is now necessary to forecast future trends in the incidence of two common complications of IHD and AH, namely acute myocardial infarction (AMI) and cerebrovascular disease, disaggregated by the urban–rural residence of patients. Table 4 presents estimates of projected incidence rates for 2025 and 2030. According to the table, the incidence of AMI is expected to be 199.8 (95% CI 80.1–319.4) per 100,000 population in residents of urban areas in 2025 and 245.5 (95% CI − 49.3 to 540.4) in 2030. The incidence of cerebrovascular disease in urban areas is projected to grow more substantially, reaching 700.6 per 100,000 population (95% CI 552.6–848.7) in 2025 and 910.0 (95% CI 533.3–1286.8) in 2030. In rural areas, the incidence of CVD complications is expected to increase more modestly, reaching levels of 121.3 (95% CI 85.1–157.6) per 100,000 and 154.8 (95% CI 70.4–239.1) for AMI in 2025 and 2030, respectively. For cerebrovascular disease, the incidence is projected to be 359.2 (95% CI 107.6–610.8) and 383.1 (95% CI − 248.6 to 1014.8) for 2025 and 2030, respectively.

**Table 4:** The projected incidence rates of acute myocardial infarction and cerebrovascular disease in urban and rural areas of Kazakhstan for the years 2025 and 2030, accompanied by 95% confidence intervals.

Incidence		Year		Model parameters		
		2025 Rate (95% CI)	2030 Rate (95% CI)	Type of model	Alpha (level)	
					t	P-value
Urban	Acute myocardial infarction	199.8 (80.1; 319.4)	245.5 (− 49.3; 540.4)	Brown	5.808	< 0.0001
	Cerebrovascular disease	700.6 (552.6; 848.7)	910.0 (533.3; 1286.8)	Holt	1.900	0.0800
Rural	Acute myocardial infarction	121.3 (85.1; 157.6)	154.8 (70.4; 239.1)	Brown	5.133	< 0.0001
	Cerebrovascular disease	359.2 (107.6; 610.8)	383.1 (− 248.6; 1014.8)	Brown	5.515	< 0.0001

*95% CI* 95% confidence interval.

**Figure 4:**
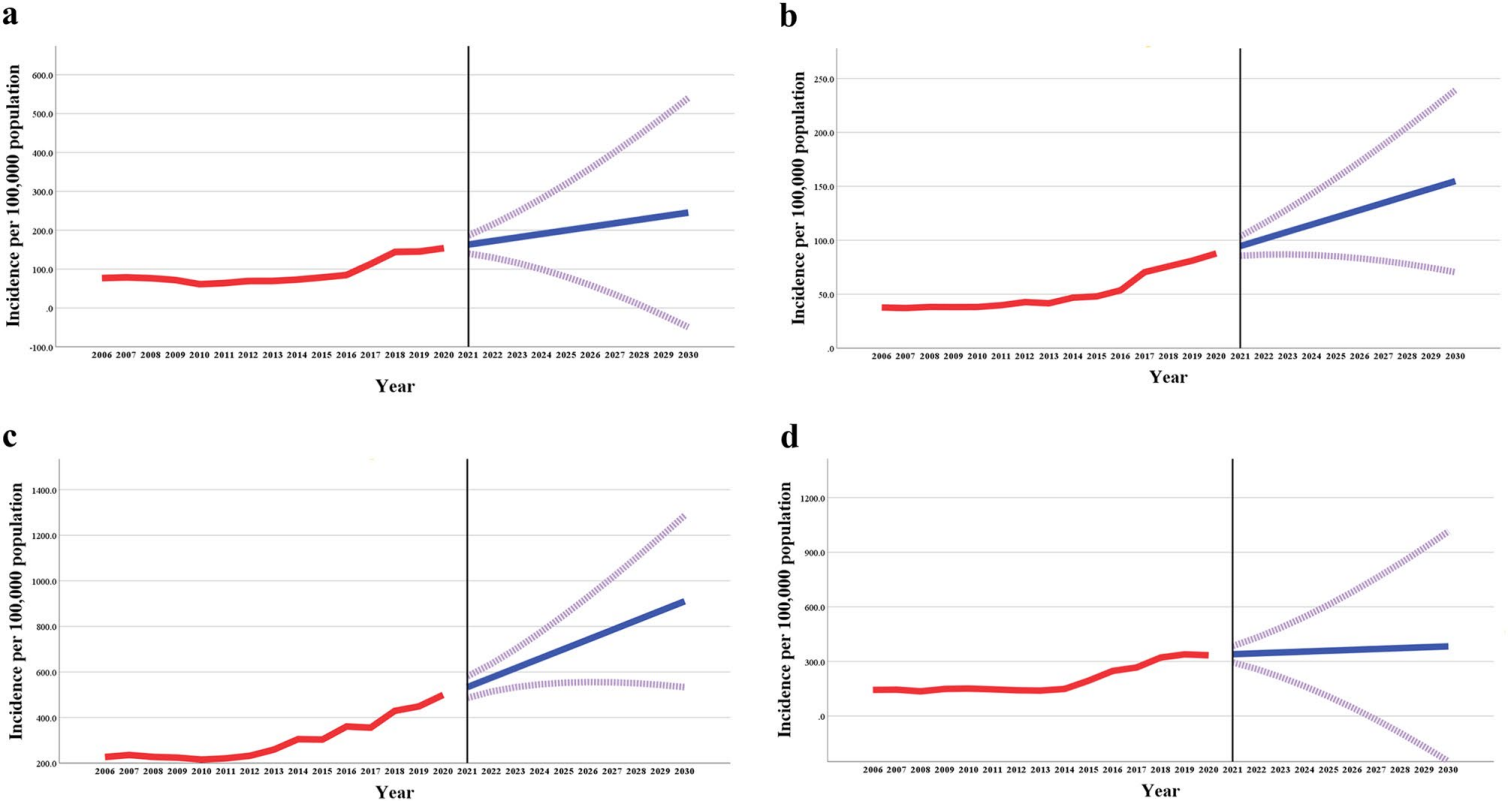
The observed and projected incidence rates of acute myocardial infarction in urban (**a**) and rural (**b**) areas, as well as the incidence rates of cerebrovascular disease in urban (**c**) and rural (**d**) areas.

Figure 4 serves as an illustration of Table 4 and depicts the observed and projected incidence rates of AMI and cerebrovascular disease in both urban and rural areas of Kazakhstan. As indicated by the figure, the incidence of both AMI and cerebrovascular disease has exhibited a steady increase between 2006 and 2020, and this trend is projected to continue. Unless appropriate measures are taken, this increasing incidence may result in a substantial disease burden.

## Discussion

This study aimed to investigate the disparities in CVD prevention strategies and incidence rates between urban and rural populations in Kazakhstan. It provides valuable insights into the preventive measures used for CVD in Kazakhstan and utilizes official statistics to support the findings. The study highlights that Kazakhstan focuses primarily on tertiary prevention of CVD by investing in hospital facilities and personnel who provide interventional cardiac and cardiac surgical treatments. However, less attention is given to secondary prevention of CVD. Of the two screening programs in place, the screening for AH appears to be more successful than the screening for IHD, which may be attributed to its relative ease. The incidence of AH has increased threefold for urban residents and 1.7-fold for rural residents from 2006 to 2020. On the other hand, the provision of screening for IHD, which involves testing total cholesterol levels with subsequent evaluation of individual risks using the SCORE chart, does not identify many new cases. Urban residents had almost a twofold increase in IHD incidence during the period of 2006–2020, while the IHD incidence in rural areas remained at the same level. The study also revealed that primary prevention strategies for CVD are largely non-existent in Kazakhstan as no policy document was identified despite a careful search. Predictive modeling for two complications of CVD, AMI and cerebrovascular disease, indicates that their incidence will continue to grow, which may overwhelm the healthcare system in the next decade. In light of these findings, Kazakhstan needs to reevaluate its approaches to CVD prevention by improving the effectiveness of IHD screening and introducing nationwide strategies for primary prevention.

Population-wide interventions offer the most promising approach to preventing disease. These interventions typically leverage mass media channels to raise awareness of key risk factors, such as promoting regular physical activity (at least 30 min per day), encouraging healthy food choices, and discouraging obesity^20^. In addition, enacting legislation to restrict smoking and reduce salt consumption represent crucial measures for primary prevention. With respect to mitigating risks associated with hypertension and hypercholesterolemia, it is more cost-effective to shift the overall population mean than relying on screening programs to identify and treat only high-risk individuals^21^. Primary prevention can also help decelerate the progression of atherosclerosis, lower the incidence of certain cancers and chronic respiratory diseases, and have a more profound impact on younger populations. By implementing these interventions, Kazakhstan may potentially reduce the likelihood of future cardiovascular epidemics, as has been observed in many developed nations from 1960 to 1990^22^.

Despite a lack of a primary prevention policy for CVD in Kazakhstan, some measures have been taken in this direction. These include implementing a smoking ban in public areas, educational institutions, healthcare facilities, catering points, public transport, workplaces, and residential entrances^23^. Coupled with the implementation of tobacco taxes, this has resulted in a decline in smoking prevalence over the past decade^24^. Nonetheless, these efforts are insufficient to effectively control the rising incidence of CVD, and more needs to be done. Kazakhstan has one of the highest levels of salt intake globally, at approximately 17 g per day, nearly four times higher than the WHO-recommended limit^25^. Furthermore, the traditional Kazakh diet is high in animal-based products such as meat and dairy, and often high in fat. Additionally, the rapid socio-economic changes in Kazakhstan over the past few decades have had an impact on the country's food culture, leading to an increase in the availability and consumption of processed foods, fast food, and sugary drinks^25^. Despite these issues, they have not received adequate attention in Kazakhstan, and failure to act promptly may result in significant economic pressures on the nation in the coming decade, due to a sharp rise in the incidence of CVD.

Globally, clinical practice guidelines are considered as the most effective health policy documents that provide comprehensive recommendations for prevention strategies based on the available evidence^26^. Despite the implementation of the World Bank project "Health Sector Technology Transfer and Institutional Reform" from 2009 to 2014^27^, which aimed to enhance healthcare quality through the adoption of internationally recognized instruments such as clinical practice guidelines, this practice did not take root in Kazakhstan's healthcare system. Instead, Kazakhstan relies on a series of legislative acts, including MoH orders and clinical protocols for diagnosis and treatment, which cover preventive services from various perspectives. However, these documents lack coordination and are largely outdated, especially the clinical protocols, which were last updated in 2016^15^. To improve the situation, Kazakhstan could consider reverting to the use of clinical practice guidelines and adapting already existing documents such as those from the World Heart Federation^28^ or European Heart Network^29^, with necessary translation and customization.

The incidence of CVD in rural areas of Kazakhstan is of interest, particularly in relation to the density of outpatient healthcare facilities. According to the statistical yearbook issued by the MoH^8^, the regions of Mangystau, Atyrau, and South Kazakhstan have had the lowest density of rural outpatient healthcare facilities over the past 5 years, and they also exhibit some of the lowest rates of IHD and AH in the country. Conversely, the regions of West Kazakhstan, Pavlodar, and North Kazakhstan had higher rates of both outpatient healthcare facilities and incidence rates of IHD and AH in rural areas above the country’s average. These findings suggest that expanding the network of rural healthcare facilities in certain regions of Kazakhstan could help to improve the identification and management of CVD. The introduction of nurse-led cardiovascular interventions could also be a viable approach, as indicated by several recent systematic reviews^30,31^.

## Study limitations

One of the main limitations of this study is its retrospective design, which created barriers to data collection and disaggregation. For instance, it was not possible to distinguish between different types of cerebrovascular disorders, such as strokes, transient ischemic attacks, and others, as they were all classified under a single diagnostic entity. Although it is reasonable to assume that strokes and transient ischemic attacks constitute a large proportion of cerebrovascular diseases, the lack of precision in diagnostic categorization may have affected the accuracy of the study's projections. However, this study also has several strengths. Firstly, it combines two approaches: a comprehensive search for health policies on CVD prevention in Kazakhstan, and an analysis of official data on CVD incidence, disaggregated by urban and rural areas. This multi-perspective approach allows for a more nuanced understanding of the existing situation, and makes the conclusions more robust. Additionally, the validity of this study is bolstered by its utilization of a sizable dataset comprised of administrative records spanning a period of 15 years and encompassing the entirety of the country. These factors lend a high degree of accuracy to the estimated results.

## Future directions

This study has significant implications for future research and policy development. Firstly, there is a clear need for studies that delve into the socioeconomic factors influencing food culture. These studies are essential for gaining a comprehensive understanding of the dietary patterns that impact cardiovascular health in both urban and rural populations and for tailoring effective interventions. Additionally, future research needs to evaluate the effectiveness of national health plans implemented in Kazakhstan during the past decade in reducing cardiovascular morbidity and mortality. This evaluation should emphasize identifying what strategies have been successful and what adjustments are needed. Furthermore, addressing the regional disparities in the distribution of healthcare facilities and the healthcare workforce, particularly in rural areas, is of utmost importance. It is imperative to identify the contributing factors to these disparities and subsequently take actions to rectify the resource imbalances.

## Conclusions

The content analysis of existing official documents indicates that over the past two decades, Kazakhstan has made significant efforts to control the increasing incidence of CVD. However, these efforts have primarily focused on tertiary prevention measures. The lack of emphasis on primary prevention measures, along with suboptimal secondary prevention, is concerning as it could lead to a rapid growth in the incidence of both CVD and its complications. To address this issue, it is essential to prioritize primary prevention measures and strengthen secondary prevention efforts, particularly in rural areas. Simultaneously, treatment and rehabilitation services should continue to be provided to patients with CVD complications. Prospective research is also necessary to gain a better understanding of the patterns of CVD and to document changes in CVD epidemiology.

### Supplementary Information

Supplementary Information 1.

Supplementary Information 2.

## Data Availability

The data used and analyzed during the current study are available from the corresponding author upon reasonable request.

## Abbreviations

AH: Arterial hypertension
AMI: Acute myocardial infarction
BP: Blood pressure
CI: Confidence intervals
CVD: Cardiovascular disease
IHD: Ischemic heart disease
MoH: Ministry of Health
NCD: Non-communicable diseases
SCORE: Systematic COronary Risk Evaluation Chart
SPSS: Statistical Package for Social Sciences
USSR: Union of the Soviet Socialist Republic
WHO: World Health Organization
