# Supplementary material for: Examining disparities in cardiovascular disease prevention strategies and incidence rates between urban and rural populations: insights from Kazakhstan
Source: Sci Rep. 2023 Nov 28;13:20917. doi: 10.1038/s41598-023-47899-8 (PMC10684854; doi:10.1038/s41598-023-47899-8)
Supplement: Supplementary file 2 — Supplementary Information 2. [file 41598_2023_47899_MOESM2_ESM.docx]

**Appendix B. Algorithms for the statistical analysis**

SPSS predictive analytics algorithms were used for the statistical analysis and were carried out in the following steps:

1. The data were prepared and arranged in Excel spreadsheet with the following variables selected for the analysis:
   1. Calendar Year (Variable’s name: Year)
   2. Incidence of Arterial hypertension in urban areas (Variable’s name: Arterial Hypertension_urban)
   3. Incidence of Arterial hypertension in rural areas (Variable’s name: Arterial Hypertension_rural)
   4. Incidence of Ischemic Heart Disease in urban areas (Variable’s name: Ischemic Heart Disease_urban)
   5. Incidence of Ischemic Heart Disease in rural areas (Variable’s name: Ischemic Heart Disease_rural)
   6. Incidence of Acute Myocardial Infarction in urban areas (Variable’s name: Acute Myocardial Infarction_urban)
   7. Incidence of Acute Myocardial Infarction in rural areas (Variable’s name: Acute Myocardial Infarction_rural
   8. Incidence of Cerebrovascular Disease in urban areas (Variable’s name: Cerebrovascular Disease_urban
   9. Incidence of Cerebrovascular Disease in rural areas (Variable’s name: Cerebrovascular Disease_rural
2. Selection of the regression model for forecasting was carried out with the help of the Expert Modeler function.
3. The incidences of the cardiovascular diseases listed under point 1 were projected for the period up to 2030 based on the incidences observed from 2006 to 2020.
4. The forecasting graphs were created and 95% confidence intervals were calculated.
5. The following parameters of the best-fit model were extracted:
   1. Type of model
   2. Incidence rates for 2025 and 2030 with 95% confidence intervals
   3. Alpha level and P-value.
